# Perturbing the developing skull: using laser ablation to investigate the robustness of the infraorbital bones in zebrafish (*Danio rerio*)

**DOI:** 10.1186/s12861-014-0044-7

**Published:** 2014-12-17

**Authors:** Carolyn T Chang, Tamara Anne Franz-Odendaal

**Affiliations:** Department of Biology, Saint Mary’s University, 923 Robie Street, Halifax, Nova Scotia B3H 3C3 Canada; Department of Biology, Mount Saint Vincent University, 166 Bedford Highway, Halifax, Nova Scotia B3M 2J6 Canada

**Keywords:** Intramembranous ossification, Morphometrics, Robustness, Lateral line canal walls, Craniofacial development

## Abstract

**Background:**

The development of the craniofacial skeleton from embryonic mesenchyme is a complex process that is not yet completely understood, particularly for intramembranous bones. This study investigates the development of the neural crest derived infraorbital (IO) bones of the zebrafish (*Danio rerio*) skull. Located under the orbit, the IO bones ossify in a set sequence and are closely associated with the lateral line system. We conducted skeletogenic condensation and neuromast laser ablation experiments followed by shape analyses in order to investigate the relationship between a developing IO bone and the formation of the IO series as well as to investigate the highly debated inductive potential of neuromasts for IO ossification.

**Results:**

We demonstrate that when skeletogenic condensations recover from laser ablation, the resulting bone differs in shape compared to controls. Interestingly, neighbouring IO bones in the bone series are unaffected. In addition, we show that the amount of canal wall mineralization is significantly decreased following neuromast laser ablation at juvenile and larval stages.

**Conclusions:**

These results highlight the developmental robustness of the IO bones and provide direct evidence that canal neuromasts play a role in canal wall development in the head. Furthermore, we provide evidence that the IO bones may be two distinct developmental modules. The mechanisms underlying developmental robustness are rarely investigated and are important to increase our understanding of evolutionary developmental biology of the vertebrate skull.

## Background

In general, amongst vertebrates, most studies investigating the development of the skull focus on the calvarial bones or jaws. Although some studies have described the growth of particular bones in the teleost skull (e.g. oral jaws, etc), less research has been conducted on the intramembranous flat bones such as the opercle or circumorbital bones. Of these, the growth and development of the opercle has been described by Kimmel and colleagues (e.g. [1] and others).

Recent studies investigating the development of intramembranous bones associated or within the eye have shown that there is a high degree of patterning involved in the development and growth of these elements [2-4]. In chicken, there is a series of neural crest derived, intramembranous bones in the sclera of the eye known as the scleral ossicles; these bones are similar to the circumorbital bones in the teleost skull in that they are also neural crest derived intramembranous bones [5-7]. The development of the scleral ossicles has been shown to be complex involving inductive tissue interactions, spatial and temporal patterning, and the involvement of multiple gene families. The scleral ossicles in chicken develop through an inductive interaction with epithelial derived conjuctival papillae that are found in direct correlation to the number of ossicles [5,8-10]. Interestingly, following ossicle inhibition, a compensatory response is observed such that the adjacent ossicle in the series expands to close the gap in the series [2,6]. Whether this is a widespread phenomenon amongst series of intramembranous bones has not been explored, until now.

In teleosts, a series of neural crest derived intramembranous bones known as the circumorbital bones surround the orbit [7,11]. The development of these circumorbital bones has already been shown to be influenced by the development of the eye in at least one teleost, the Mexican tetra *Astyanax mexicanus* [3]. In this study, following embryonic lens removal, the eye regressed and the resulting adult skeleton was affected such that the supraorbital and suborbital bones of this bone series enlarged into the orbital region. It was hypothesized that the effect was caused by changes in neural crest cell migration and/or mechanical forces due to eye regression [3]. This study highlights the interactions between the soft tissues of the eye and the hard tissues surrounding the eye (i.e. the circumorbital bones) and demonstrates the existence of developmental plasticity within these bones.

The infraorbital (IO) bones are a component of the circumorbital series and are located around the underside of the orbit. The IO bones are named, infraorbital one through five, according to their position in the skull from anterior to posterior [11]. The function of the IO bones has not yet been directly investigated; however with such a close association to the eye, these bones likely serve a protective or supportive role. The development of the five IO bones is complex and these bones mineralize late in the sequence of zebrafish craniofacial ossification [4,11].

**Figure 1:**
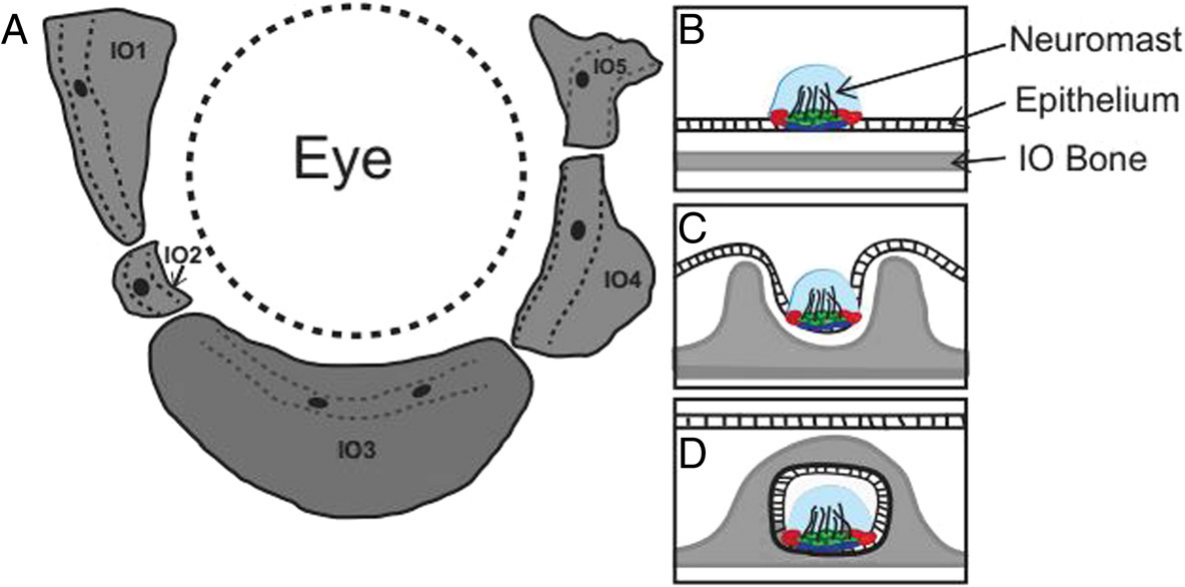
**Relationship between infraorbiral bones and cranial neuromasts.** **(A)** A schematic of the IO bones on the left side of the zebrafish skull. Dashed lines indicate lateral line canals and black dots are primary canal neuromast mechanosensory receptors. **(B-C)** During development the primary neuromasts are enclosed in the bony lateral line canal of the underlying IO bone. Neuromast enclosure occurs in stages as described by Webb and Shirey (2003) [13]: **(B)** a canal neuromast is present on the epithelium overlying an IO bone, **(C)** the neuromast sinks into an epithelial groove and bony canal walls mineralize outwards on either side of the neuromast; **(D)** and finally an epithelial canal roof forms followed by the enclosure of bony canal walls which approach one another. **B**-**D** are modified from Webb and Shirey (2003) [13].

All five IO bones consist of a flat bone component as well as a bony canal which houses part of the cranial lateral line. The functional unit of the lateral line system is the neuromast, a mechanosensory receptor organ, similar to that of the stereocilia of the inner ear of humans. At the core of the neuromast are mechanosensory hair cells, which are innervated by sensory neurons [12]. The hair cells are surrounded by both support and mantle cells, the latter of which are thought to secrete the cupula that surrounds them (Figure 1) [12]. Two kinds of neuromasts have been described in teleosts [13]. Presumptive (primary) canal neuromasts are found enclosed within the lateral line canals while superficial (secondary) neuromasts are found in the skin [14,15]. It is the primary neuromasts that are present within the IO lateral line canals. Webb and Shirey [13] described four developmental stages of canal neuromast development in the zebrafish head (summarized in Figure 1B-C). The lateral line canals generally form between 10.5 mm SL (juvenile) and adulthood (reproductive maturity; 4 months of age) [4,13].

Since skeletogenesis involves induction from epithelia [9,13,16-18] and since placodes (including neuromasts) arise from epithelia (e.g. [14,15] and are in close association with circumorbital bones, it has been proposed and debated that placode-derived neuromasts have inductive potential with regards to inducing bony canal wall formation [13,16,19-21]. One hypothesis put forth was that the neuromasts induce the formation of the bony canal since they form prior to the formation of the canals in many teleosts (reviewed in [16]). However, in some species (e.g. *Ophicephalus punctatus* Bloch), the bony canals are present prior to neuromast formation (reviewed in [16]). Earlier researchers argued that the neuromasts do not directly induce canal wall ossification, but change the mechanical [17] and biochemical [18] environment which triggers ossification. A large portion of this literature was based on inferences made from observations. Only one study has directly tested this hypothesis in the cranial lateral line system of teleosts. DeVillers (cited in [13]) ablated the neuromasts of the supraorbital canal in salmonids and observed abnormal morphogenesis of the frontal bone. More recently, in zebrafish, Webb and Shirey [13] investigated the development of cranial lateral line canals and neuromasts in a growth series via SEM and histology; they again hypothesized that neuromasts may induce the bony lateral line canal walls during the second stage of canal development when the neuromast sits in an epithelial groove for an extended period of time [13], however no direct experimental evidence was provided. Since this work, little further knowledge has been gained on the relationship between bony lateral line canals and their associated neuromasts. In the trunk lateral line system, however, interactions between posterior lateral line and scale formation have been previously tested and results suggest that lateral line cells play an important role in canal formation in killifish (*Fundulus heteroclitus*) [19] and goldfish (*Carassus auratus*) [20]. Also, recently in zebrafish (*Danio rerio*) Wada et al. [21] investigated the development of the trunk lateral line system. Similar to killifish and goldfish, lateral line cells are required for the formation of canals in the zebrafish posterior lateral line. These results suggest that there are mutual interactions during development between the sensory cells of the lateral line and the hard connective tissues of the scales.

Here, we investigate the development and growth of the zebrafish infraorbital bones and the relationship between these bones and the lateral line canal neuromasts in the head. These relationships will be elucidated through the completion of two main objectives. First, we aim to investigate the developmental robustness of the IO bones and to determine whether individual developing infraorbital bones affect the development and/or growth of neighbouring IO bones. Developmental robustness refers to the ability for a phenotype of an organism to remain on a developmental pathway despite variation in or perturbation of an environmental or genetic parameter [22,23]. We conducted targeted laser ablation of single IO skeletogenic condensations in the IO series and then analysed the results over a period of three months. We hypothesize that IO series is robust and following laser condensation ablation some recovery of the element will occur in order to maintain the IO series. If the IO series is not robust then limited or no recovery of the IO bones will follow condensation ablation. We also hypothesize that individual developing IO bones will affect the development and/or growth of neighbouring IO bones based on work in the chicken scleral ossicle system. Second, we aimed to directly test whether developing canal neuromasts influence the development of the bony canal walls surrounding them through targeted ablation of selected IO primary neuromasts prior to IO canal formation. We hypothesize that, similar to the trunk lateral line canals, developmental interactions between the sensory cells of the neuromast and the hard tissue of the infraorbital bone canals are required for formation of the infraorbital lateral line canal.

## Results

To determine the role that individual bones play in the development of adjacent bones in the IO series, we compared short and long-term results of targeted skeletogenic condensation ablation experiments. We analysed the targeted element, control elements (on the contralateral side) and control fish via gross morphology and shape analyses. We also analysed the effect on the adjacent bones. Short-term results were collected on anesthetized juvenile specimens over the two-month period immediately following skeletogenic ablation, whereas long-term results were collected once specimens had reached adulthood (3 months) and were fixed. This type of long term study involving ablation at juvenile stages and analyses at multiple time-points during development to adulthood is rarely conducted because fish naturally die during the grow-out phases. Our second objective was to test whether developing canal (primary) neuromasts influence the development of the bony canal walls surrounding them through targeted ablation of neuromasts prior to IO canal formation. For these analyses additional fish were used; these fish underwent neuromast ablation at larval and juvenile stages to determine whether neuromast ablation affects infraorbital bone development. For all experiments, we targeted our analyses to infraorbital three through five which are second (IO3 and IO5 develop concurrently) and third (IO4) in the IO bone series to form. Skeletal condensation ablations were performed on IO5 with follow-up on IO4-IO5 to determine whether the neighbouring bone was affected. Neuromast ablations were performed on IO3-IO5 canal neuromasts with follow-up on IO3-IO5 bones.

### The infraorbital condensation recovers from ablation and the series is unaffected

**Figure 2:**
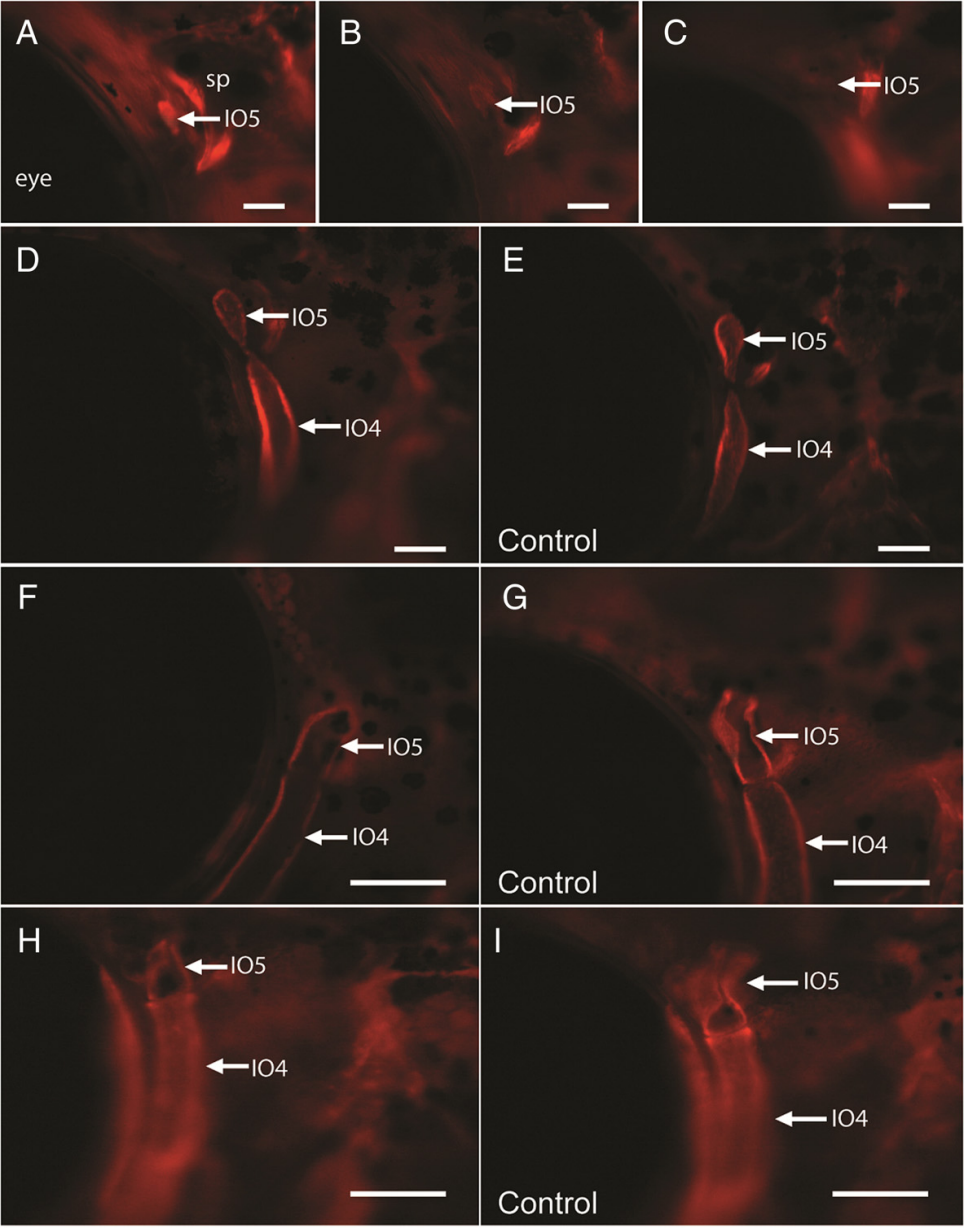
**Laser ablation of the IO5 condensation in a transgenic juvenile osterix:mcherry zebrafish. (A)** Before ablation IO5 condensation occurs anterior to the sphenotic (sp); **(B)** immediately after ablation the IO5 condensation is not present and does not fluoresce; **(C)** 24 hours post-ablation the IO5 condensation is still not present. The arrow in **A**-**C** indicates the posterior edge of IO5 condensation, the dark space anterior to the element is the eye. The other two bright regions are the anterior and anterodorsal edges of the spenotic (sp) which begins to ossify shortly before IO5. **(D)** by 4 days post-ablation a IO5 condensation has reformed (arrow) and touches the IO4 condensation unlike the control-side **(E)** in which the IO4 and IO5 are separate condensations; **(F)** the IO5 continues to grow at 2 weeks post-ablation and is still closely associated with IO4; it appears less developed (lacking canal walls) compared to the control side shown in **(G)**. Two months following IO5 condensation ablation, the IO5 element is morphologically different to the control-side, compare **(H)** the ablation side to **(I)**, the control side. The ablated side **(H)** lacks the anterodorsal flat-wing extension and canal walls are not approaching each other compared to control-side **(I)** Lateral view, anterior is to the left, Scale bars are **(A-C)** 50 μm and (D-I) 25 μm.

To investigate the role that an individual IO bone plays in the development of the IO series the IO5 condensation (the second smallest IO bone) was ablated using the nitrogen laser ablation system previously established by Berns et al., [24] and used to target vertebrate cells (e.g. in axolotl: [25,26]; zebrafish: [27,28]. In the short term, immediately following ablation, no visible signs of the IO5 condensation is present (N = 8) (Compare Figure 2A and B). Similarly, at 24 hours post-ablation the ablated condensation is still not visible (Figure 2C); however, by 4 days post-ablation the IO5 condensation has reformed (N = 8) (Figure 2D and E). This IO5 condensation is smaller when compared to the IO5 condensation on the control side (N = 7) (Figure 2D). In addition, it is closer to the adjacent IO4 bone and appears to pinch off from the IO4 element via a narrow bridge of bone tissue (Figure 2D arrow). By two weeks post-ablation the difference in size between the experimental and control-side IO5 elements is still evident (Figure 2F to G). Overall, the recovered IO5 appears smaller and truncated in the dorsal-ventral length compared to the control side. At this age, a morphological difference is also observed in the lateral line canal wall; the recovered IO5 bone lacks distinct canal walls whereas in the control IO5 element, the canal walls approach one another. Similarly, at two months, the reformed IO5 element appears morphologically different from the control IO5 in three out of the four specimens (Figure 2H). Unlike control IO5 elements (control-side: N = 4; control specimen: N = 5), the reformed IO5 element in affected specimens (3/4 as above) does not possess the prominent bend in the canal region nor the flat, wing-like extension (Figure 2H and I). The delay in canal wall formation is also still apparent. This difference persists into the long-term, such that by adulthood (19.0-22.0 mm SL), the recovered IO5 bone is still truncated, has a less bent canal as well as a smaller wing-like extension compared to the control side and unablated specimens. In the fourth specimen, the IO5 bone is similar in shape and size to control IO5 bones and possess both the flat-wing aspect and outgrown canal walls. No difference in the development of the other IO bones (IO1-4) in the series was observed when compared to the time-points we recently described [4,11]. These results demonstrate that (i) an infraorbital condensation can recover from ablation within 1-4 days; (ii) that the recovered condensation develops into smaller bones when compared to controls, and (iii) that the adjacent bones in the series are unaffected. We hypothesize that this recovery mechanism is similar in all the IO bones in the series; however, this response remains to be tested for each individual IO bone.

**Figure 3:**
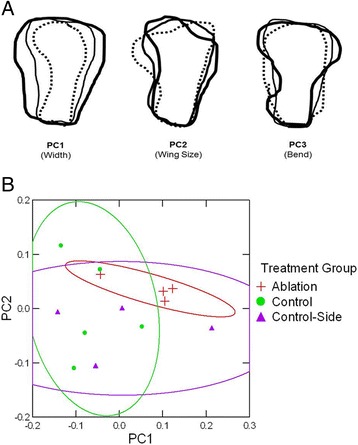
**Variations in the shape of the IO5 bone in adults accounted for by the 1st, 2nd, and 3rd principal components.** IO5 condensation ablation (red plus sign), control-side (purple triangle) and control (green circle) specimens. **(A)** The contour variance of each principal component is shown; broken line represents the average -2 SD from the mean, thin solid line represents the mean and the thick solid line, indicates +2 SD from the mean where SD stands for standard deviation. **(B)** Scatterplot of PC1 versus PC2 for the IO5 condensation ablation experiment. 95% confidence interval ellipses are also shown with the same treatment group colours.

Following these analyses and to further quantify the observed gross morphological differences in adult IO5 shape after ablation, a shape analysis was performed using the SHAPE software program. Shape analysis enables a quantitative evaluation of biological contour shape based on Elliptic Fourier Descriptors (EFD) [29]. The contours of ablation, control-side and control IO5 elements were compared in this analysis (Figure 3A). The first three principle components (Table 1) cumulatively contribute to over 70% of the total variance in IO5 contour. Following visualization in PrinPrint, the morphological meaning associated with each principal component was delineated. The first principle component (PC1) corresponds to the relationship between the anterior-posterior width compared to the dorsal-ventral length and accounts for 37% of the total variance (Figure 3A: PC1). The second principle component (PC2) corresponds to the size of the flat, wing-like aspect of the bone and accounts for 22% of the variance (Figure 3A: PC2). Finally, accounting for only 14% of the variance is the third principle component (PC3), which describes the severity of the lateral line canal bend (Figure 3A: PC3). A scatter plot (Figure 3B) of the first two principal components shows a tight cluster of three of the four ablation specimens with the fourth ablation specimen located amongst both the control and control-side specimens. These three ablation specimens have PC1 scores corresponding to a wider element (Figure 3: PC1 bold outline), as well as PC2 scores that correspond to truncated (dorsal-ventral) elements with smaller flat wing-like extensions (Figure 3: PC2 bold outline). Control elements have PC1 and PC2 scores related to mean (narrow line) and negative scores (dashed outlines). To analyze this observed grouping, 95% confidence ellipses were added to the scatter plot (Figure 3B). The confidence ellipses of all treatment groups overlap to some extent; however three of the four ablation specimens form a tight cluster away from all but one control-side specimen. This overlap is most likely due to the small sample size and occurrence of outliers. No clusters are observed along PC3 as ablation, control-side and control IO5 elements were evenly distributed amongst one another along this axis (not shown). In summary, the shape analysis results reveal that ablated condensations develop into IO5 bones in adults and are differ in morphology from control-side and control (unablated) IO5 elements suggesting that skeletogenesis recovers after ablation.

**Table 1:** **Summary of IO5 condensation ablation experiment shape analysis and principal component analysis results for the elliptical Fourier descriptors of adult element contour shape**

		**PC axis**			
		**PC1**	**PC2**	**PC3**	**PC4**
IO4 shape analysis	Eigenvalue*	11.8	1.98	1.48	0.63
	Proportion variance (%)	68.64	11.54	8.63	3.66
	Cumulative variance (%)	68.64	80.19	88.82	92.48
	Shape described	Flat-wing size	Element width	Flat-wing ventral edge	Flat-wing dorsal edge
IO5 shape analysis	Eigenvalue*	8.56	4.98	3.28	2.83
	Proportion variance (%)	37.42	21.75	14.32	12.37
	Cumulative variance (%)	37.42	59.18	73.50	85.87
	Shape described	Element width	Flat-wing size	Canal bend	Ventral edge shape

*Read eigenvalue as χ x 10^-3^.

Eigenvalues, proportion of variances, and shape character described for PC1-4 for adult IO4 and IO5 elements of ablation, control, and control-side adult specimens.

Furthermore, in order to determine whether the ablation of IO5 condensation affected the neighbouring adult IO4 element, additional shape analysis on IO4 was conducted. The contours of ablation, control-side and control IO4 elements were compared (N = 13 contours). The first two principle components cumulatively contribute to over 80% of the total variance (Table 1). As for IO5, following visualization in PrinPrint, the morphological meaning associated with each principal component was deduced. The first two principal components correspond to the flat, wing-like extension of the IO4 bone. Scatter plots of the first two principal components show no specific groupings of ablation and control specimens (not shown) indicating that IO5 condensation ablation does not affect the shape of the adjacent IO4 elements. That is, the rest of the IO bone series is normal.

### Infraorbital canal neuromasts recover from targeted ablation

**Figure 4:**
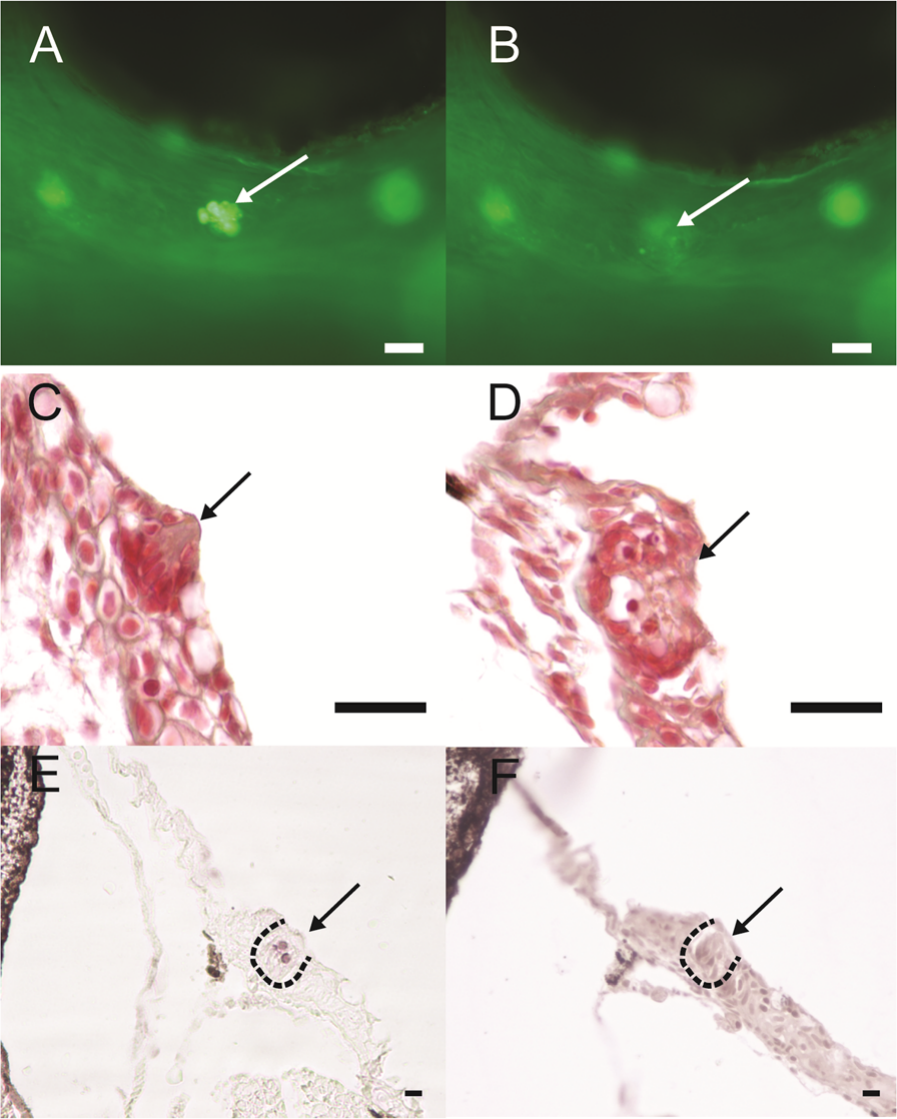
**Representative figures of targeted neuromast laser ablation on infraorbital three canal neuromast. (A)** Before and **(B)** after neuromast laser ablation in live zebrafish specimens stained with FM1-43. Masson’s trichrome stained cross-section of neuromast of **(C)** control-side and **(D)** post-ablation neuromasts. **(E and F)** TUNEL stained cross-section of ablated neuromasts **(E)** and control side **(F)**. Arrow indicates neuromasts. Scale bars are **(A and B)** 25 μm and **(C-F)** 10 μm.

In order to determine whether IO canal neuromasts can recover from ablation and if there are subsequent effects on IO bone canal wall formation, IO neuromasts were ablated at both larval and juvenile time-points in the IO3- IO5 bones (Figure 4A-D shows representative images of neuromast ablation and recovery for all IO neuromasts ablated). In larval fish, immediately following the IO3 and IO4 neuromast ablation, there are no visible signs of the ablated neuromast nor are there signs of re-growth 24 hours post-ablation (N = 20) (Figure 4A-B). At 5 days post-ablation the neuromast reformed, however it appears smaller (with fewer cells) compared to before ablation (N = 20). To determine whether IO neuromasts of juvenile specimens would respond in the same way, IO3 and IO5 canal neuromasts were ablated at a time point preceding the suggested time (ablation at 10.0 mm SL) of neuromast induction of canal wall ossification [13]. Similarly, in juveniles, immediately following the IO3 neuromast ablation there is no visible sign of the ablated neuromast (N = 10) (Figure 4A-B). Also 24 hours after this, IO3 neuromast ablation at juvenile stages (N = 10), no neuromasts are observed, however by four days post-ablation the neuromast reformed and is similar in morphology to controls. The same results were observed in IO5 (N = 20) neuromast ablation experiments. These results demonstrate that canal neuromasts can recover from targeted ablation and that the recovery rate is different depending on the age of the fish at the time of ablation.

In order to confirm that tissues surrounding the targeted neuromast were not damaged during the ablation procedure we conducted histological and TUNEL staining. One hour following ablation, the neuromast shows evidence of damage but the surrounding tissues appear normal in section (compare Figure 4C, D). TUNEL staining conducted three days post-ablation shows that cell death is observed in the neuromast but not in surrounding tissues (Figure 4E). This finding agrees with previous studies that have used this technique [27,28]. No evidence of cell death was observed in controls (Figure 4F).

### Neuromast ablation affects canal wall mineralization but not bone shape

**Figure 5:**
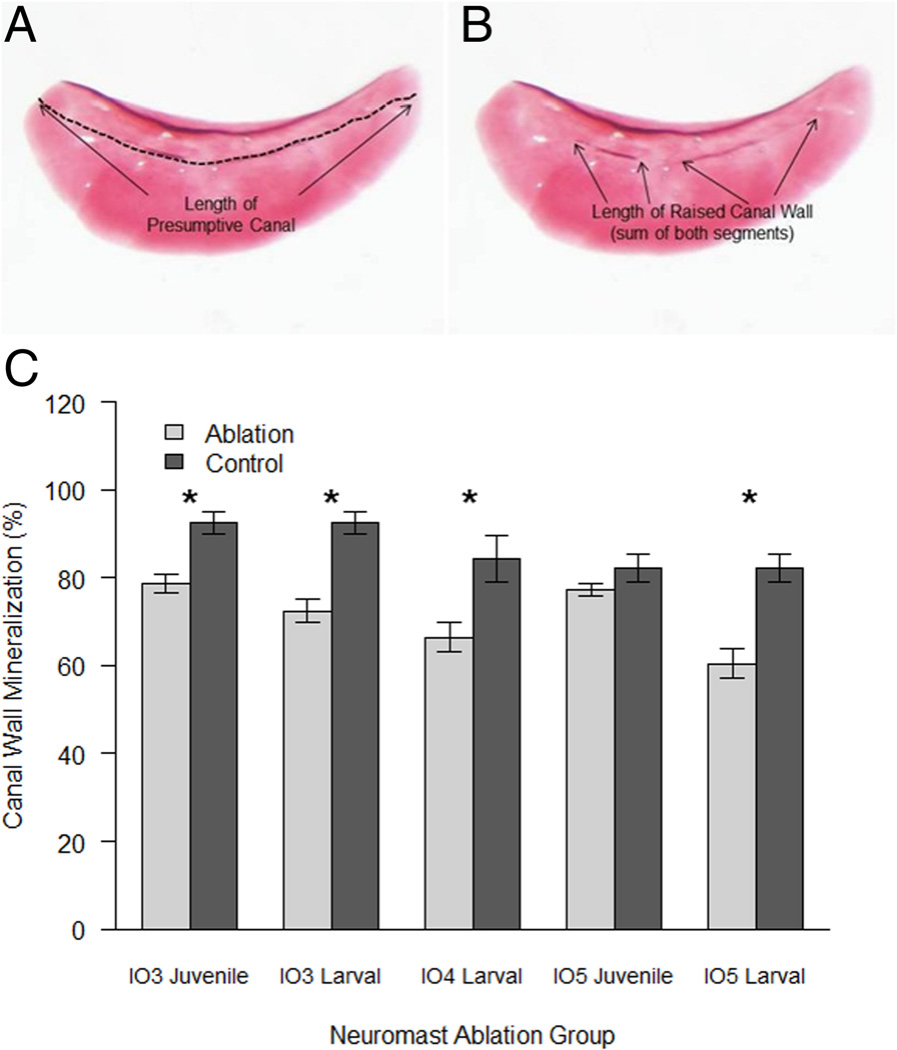
**Percentage of canal wall mineralization following infraorbital canal neuromast ablation in adults. (A)** Diagram showing the total length of canal (dashed line) and **(B)** length of mineralized canal wall (arrows) in an adult infraorbital three. **(C)** Bar graph summarizing the percent of the IO canal length that formed a mineralized wall (y-axis). Error bars show standard errors. Significance is indicated by asterisk where ablation groups differed from controls (no ablation): p < 0.05 in larval specimens for IO3 (t(23) = 0.329), IO4 (t(22) = 4.19) and IO5 (t(21) = 4.69) bones. Juvenile IO3 ablation specimens were also significantly different (t(18) = 4.215, p < 0.05).

To determine whether canal neuromasts are involved in the induction of the bony canals that enclose the lateral line system of the infraorbital bones, the extent of canal closure was measured in ablation and control specimens. Canal closure was significantly less in adult specimens that had received larval neuromast ablation compared to controls (IO3 bones: t(23) = 0.329, p <0.05; IO4 bones t(22) = 4.19, p < 0.05; and IO5 bones t(21) = 4.69, p < 0.05) (Figure 5). The extent of canal closure was also significantly less in adult specimens that had received the juvenile neuromast IO3 neuromast ablation compared to controls [IO3: t(18) = 4.215, p < 0.05]. Surprisingly, there is no significant difference when the neuromast of IO5 bones were ablated at the juvenile stage. This is likely due to the differences in the timing of IO3 versus IO5 bone formation [4], or because IO5 has a smaller canal compared to IO3. Overall, these results suggest that there is a relationship between neuromasts and lateral line canal wall ossification and that the role of neuromasts in canal wall outgrowth may be temporally dependent on the stage of bone development at the time of ablation or on the length of the canal.

To analyze the shape of IO bones between neuromast ablation and control specimens another shape analysis was performed using the contour of the entire element (canal and flat wing portion). These analyses were performed on for each ablation experiment separately (i.e. larval IO3 and IO4 neuromast ablation, juvenile IO3 neuromast ablation and juvenile IO5 neuromast ablation). For the larval neuromast ablation experiment the contour shape of IO3-IO5 were compared between ablation and control specimens. No specific groupings between ablation and control specimens were observed (not shown). For all IOs analysed, the first three principal components described at least 85% of the variation in shape (Table 2). The first principal component accounts for more than 50% of the variation and corresponds to the outgrowth of the flat-wing aspect of elements. For the juvenile IO3 neuromast ablation experiment the contour of IO3 elements were compared between ablation and control specimens. The first three principal components account for more than 90% of the variance in element shape (Table 3). The first principal component accounts for more than 40% of this variation and represents the taper direction of the flat-wing aspect of the element. Analysis of the juvenile IO5 neuromast ablation specimens involved comparing the contour of IO5 elements between ablation and control specimens. Results from this analysis show that the first three principal components explain more than 85% of the element shape variance (Table 3). The first principal component accounts for more than 40% of the variance and corresponds to the bend in the lateral line canal. For both juvenile ablation experiments, when plotted, no specific groupings are observed between ablation and control specimens (not shown). Altogether, results from the neuromast ablation experiments demonstrate that canal wall outgrowth is affected by neuromast ablation; however the overall element shape is not affected.

**Table 2:** **Summary of larval neuromast ablation experiment shape analysis and principal component analysis results for the elliptical Fourier descriptors of adult element contour shape**

		**PC axis**			
		**PC1**	**PC2**	**PC3**	**PC4**
IO3 shape analysis	Eigenvalue*	2.07	1.09	0.37	0.16
	Proportion variance (%)	50.60	26.56	9.15	3.90
	Cumulative variance (%)	50.60	77.15	86.30	90.20
	Shape described	Dorsoventral depth	Taper direction	Dorsal-edge curve	Flat-wing size
IO4 shape analysis	Eigenvalue*	12.11	5.14	3.24	1.03
	Proportion variance (%)	50.53	21.50	13.56	4.32
	Cumulative variance (%)	50.53	72.13	85.69	90.01
	Shape described	Flat-wing size	Canal curvature	Taper direction	Flat-wing dorsal edge
IO5 shape analysis	Eigenvalue*	8.89	2.50	0.91	0.73
	Proportion variance (%)	63.31	17.84	6.47	5.20
	Cumulative variance (%)	63.31	81.15	87.63	92.83
	Shape described	Flat-wing size	Element width	Canal bend	Flat-wing dorsal edge

*Read eigenvalue as χ x 10^-3^.

Eigenvalues, proportion of variances, and shape character described for PC1-4 for adult IO3, IO4 and IO5 elements of ablation, control, and control-side adult specimens.

**Table 3:** **Summary of juvenile neuromast ablation experiment shape analysis and principal component analysis results for the elliptical Fourier descriptors of adult element contour shape**

		**PC axis**			
		**PC1**	**PC2**	**PC3**	**PC4**
IO3 shape analysis	Eigenvalue*	5.2	2.17	0.41	0.1
	Proportion variance (%)	44.52	38.64	7.40	3.44
	Cumulative variance (%)	44.52	83.16	90.57	94.01
	Shape described	Taper direction	Dorsoventral depth	Dosral-edge curve	Flat-wing size
IO5 shape analysis	Eigenvalue*	11.74	8.37	4.92	1.07
	Proportion variance (%)	40.83	29.01	17.13	3.71
	Cumulative variance (%)	40.83	69.84	86.96	90.68
	Shape described	Canal bend	Dorsal-edge shape	Flat-wing size	Flat-wing dorsal edge

*Read eigenvalue as χ x 10^-3^.

Eigenvalues, proportion of variances, and shape character described for PC1-4 for the IO3 elements of IO3 juvenile ablation experiment control, and control-side adult specimens as well as for the IO5 elements of IO5 juvenile ablation experiment control, and control-side adult specimens.

## Discussion

This study investigates the development of the neural crest derived, intramembranous, infraorbital bones of the zebrafish (*Danio rerio*). We conducted a series of skeletogenic condensation and neuromast laser ablation experiments, in order to investigate both the development of bones in a series and the highly debated inductive potential of canal neuromasts. The results of this study highlight the developmental robustness of the infraorbital bones in zebrafish.

### Infraorbital bone series can rescue from ablation and exhibit modularity

In order to investigate the development of intramembranous bones in a series, we ablated IO5 condensations at 8.0-11.0 mm SL. We show that five days following ablation, the ablated condensation re-appears and appears to bud off the adjacent IO4 condensation (Figure 2D). Interestingly, IO4 is the next element to undergo osteogenesis in the IO series. This suggests that the recovery mechanism observed in the IO series maybe similar to that observed in the chick scleral ossicle system [2,6] where removal of one bone in the series results in expansion of the next bone in the series. This observed recovery mechanism of a series of intramembranous bones might be shared by all vertebrates and is very intriguing. Alternatively, the reformed IO5 element may arise due to increased osteoblast proliferation and differentiation of a few cells as shown by Willems et al., [30]. In this study [30], osteoblasts were targeted and ablated using a cytotoxic agent and following ablation new osteoblasts gradually appeared suggesting that the skeletogenic cell lineage may possess regenerative properties in medakas. It is possible therefore that in our experiments a similar regenerative mechanism of osteoblasts may be involved following condensation ablation. Altogether, bone recovery following ablation presents an interesting scenario involving natural developmental rescue mechanisms that exist in the fish skeletal system. More research is needed to enhance our understanding of these mechanisms. These long-term experiments are time consuming and therefore rarely conducted yet can be quite informative as they provide valuable insight into relationships between the development of hard tissues to one another as well as interactions between soft (i.e. neuromasts) and hard tissues. Understanding these mechanisms will enable us to understand the robustness of skeletal elements and their resistance to experimental perturbation and in nature as a result of environmental disturbances.

To evaluate adult morphology of the IO bone after ablation, we conducted a bone shape analysis. Our results show that the recovered adult IO5 element is truncated (smaller and lacking the flat, dorsal projection) and the canals are shorter. It is known that multiple signalling events are involved in bone shape. For example, the frontonasal skeleton and beak in the chicken are formed through multiple, temporal- and spatially-specific changes of proliferative zones [31]. The outgrowth of the chick frontonasal mass is directed by fibroblast growth factors from the ectodermal zone [32], whereas, the width of the beak has been shown to be regulated by the morphogen *BMP-4* [31]. Indeed, we have previously shown that two phases of IO mineralization are present in the zebrafish IO series. Some IO bones (e.g. IO1) form the flat bone aspect of the element prior to forming a canal, whereas others (e.g. IO2-5) form the canal aspect prior to the outgrowth of the flat, wing-like extension [4]. These data suggest that temporal and spatially-distinct patterning events may exist in IO bone development. The reason for the resulting truncated IO5 element after ablation may be because it ossifies slightly later than controls because it first has to recover from ablation. This delay could then affect the ossification and outgrowth of the dorsal aspect of the element. The condensation ablation data provided here further supports this hypothesis. That is, temporally distinct signalling events are involved in the development of the flat, wing-like extension of the bone that are distinct from those for the bony canal aspect of the IO bone (i.e. two distinct developmental modules may exist).

Furthermore, we show that despite appearance that the recovered IO5 buds off IO4, no difference in IO4 shape in adults were found via shape analyses. The mechanism explaining this phenotype requires further investigation and is beyond the scope of the current study. Although our sample size was limited for this part of the study, our data provides interesting insights into how intramembranous bones respond to perturbation over the long term.

### Ablated neuromasts recover faster from ablation in juveniles

Various methods have been used to ablate neuromasts in vertebrates [zebrafish: aminoglycoside antibiotics (i.e. neomycin) [33,34] and copper sulphate [35]; Mexican tetra (*Astyanax mexicanus*): adhesive tape [36,37]; salamander: laser ablation [25]; axolotls: laser ablation [26]]. Following ablation, the recovery or regeneration of neuromasts has been reported in amphibians and zebrafish (salamander: [25]; axolotls: [26]; zebrafish: [33-35]. The suggested mechanism of this neuromast recovery is that new hair cells arise via proliferation from the neuromast support cells (salamander: [25]; axolotls: [26]; zebrafish:[33]. The recovery of neuromasts following ablation in our study is also therefore likely to have occurred through this mechanism.

The severity of the effect of neuromast ablation appears to correlate with the duration of the recovery period as well as the mechanism of recovery [35]. When zebrafish were exposed to mild levels of copper sulphate only mature hair cells were damaged, while support cells were unaffected. This incomplete neuromast ablation resulted in regeneration in less than 24 hours, and these new hair cells developed from non-dividing support cells. However, when exposed to a higher dose of copper sulphate the whole neuromast including the support cells was ablated and recovery did not occur until after more than 24 hours. When recovery did occur, it involved proliferation of pre-mitotic precursor support cells. These mitotically active support cells were located at the base of the neuromast underlying the neuromast hair cells and the mature support cells. In our study, involving targeted ablation of individual neuromasts, no neuromast recovery was observed until after 24 hours post-ablation indicating that similar to the copper sulphate experiment, laser ablation likely affected the entire neuromast structure (mature hair cells and mature support cells) either directly or indirectly. We hypothesize that the recovered neuromast probably reforms via proliferation of pre-mitotic precursor support cells.

By ablating neuromasts at different times during growth of the fish, we show that neuromast recovery occurs faster in juvenile specimens compared to larval specimens. One reason for this observation might be that hair cells are added to neuromasts over a life span [38,39], and therefore there are too few premature precursor cells present at larval stages for rapid recovery. In addition, larval-fish that are dependent on their yolk for nutrients may have limited energy resources. Further research is required to fully explain this observed difference in recovery rate between juvenile and larval fish.

### Neuromast ablation affects canal wall mineralization but not IO shape

To evaluate the effect of ablation on canal wall development, we analysed canal wall outgrowth in ablation and control adult specimens. We show that following neuromast ablation, the outgrowth and closure of mineralized canal walls is significantly affected in larval and juvenile ablations supporting our hypothesis that neuromasts are involved in canal wall mineralization. Surprisingly, we found that even when the neuromast ablation was conducted at the larval stage, 3-4 weeks prior to the hypothesized period of neuromast induction of canal walls [13], the amount of canal wall mineralization in the resulting adult bones was significantly decreased. Two potential mechanisms could explain these results. First, the neuromast ablation caused a decrease in inductive signals for canal wall formation due to the time required for the ablated neuromast to recover and this delay then translates into a lost period of signalling for mineralization. Alternatively, the regenerated neuromast has a weaker inductive potential than the original neuromast and the inductive signal might not be able to travel as far in the tissue. For example, the observation of decreased canal wall mineralization in juvenile IO3 neuromast ablation specimens, but not in IO5 ablation specimens may be because IO3 has a longer; a decrease in the strength of the inductive signal in recovered neuromasts would therefore affect IO3 more. Although, more research is required to fully understand the role neuromasts play in IO canal mineralization, this study shows that IO canal wall development is affected by canal neuromasts.

Finally, in the neuromast ablation experiments, the overall shape of the adult ablated IO bones was not affected suggesting that the flat bone portion is independent of the canal wall portion in these bones and supports a bi-modular developmental system. We previously showed that IO bone development has two phases of bone development, a canal phase and a flat-wing phase, either of which can occur first [4]. We further hypothesized that infraorbital development may involve two developmental modules. Modularity is the word coined to describe when parts of an organism are closely integrated and connected, more so with one another than with neighbouring modules (e.g. [40] and others). Modules are therefore also hierarchical in nature. Modularity in the cranial lateral line system of teleosts has recently also been discussed by [41]. Recently, Webb et al. [42] showed that in Lake Malawi cichlids, the morphology of the lateral line canals and the dermal bones in which they are found can evolve independently of each other [42]. Taken together, this data further supports the idea that the lateral line canal system of teleosts is modular.

In summary, we explored and tested the relationship between bones in the IO series and the lateral line canal neuromasts. Our results provide direct evidence that canal neuromasts play a role in the formation of canal walls. We further hypothesize that neuromast recovery occurs through proliferation of support cells resulting in a delay or change in the inductive potential of the recovered neuromast. More research is required to fully elucidate the inductive mechanism and extent of neuromast involvement in canal wall mineralization.

## Conclusions

In conclusion, the IO series of bones is a developmentally complex system that has remained together in skull ontogeny throughout evolution. Clearly there must be developmental mechanisms in place to have maintained this bone series throughout 450 million years of evolution [43,44]. The results of this study highlight both the complexity and robustness of these bones. The mechanisms of developmental robustness in skeletogenesis have not received much attention in the literature yet these studies are critically important to increase our understanding of intramembranous bone patterning and growth. The canal neuromasts were shown to play a role in canal wall mineralization indicating that the IO series is also an ideal system to further elucidate the interaction between hard and soft tissues.

## Methods

### Zebrafish

Wild-type zebrafish (*Danio rerio*) embryos were bred in our laboratory from AB stocks sourced from the Zebrafish International Research Center (ZIRC) while a limited number of transgenic osx:CFP zebrafish embryos were provided by Dr. Shannon Fisher (University of Pennsylvania). Wildtype zebrafish were used for all neuromast studies while the transgenic fish were used for the condensation ablation experiments. All fish were reared following standard conditions to time-points required for the experiments in this study (28.5°C on a 12 hour light, 12 hour dark cycle). Fish were housed at Mount Saint Vincent University, Halifax Nova Scotia, Canada. All protocols follow Canadian Council on Animal Care guidelines, which were annually reviewed by the SMU-MSVU Animal Care Committee. Anaesthesia through immersion in 0.01% MS222 (ethyl-3-aminobenzoate methane sulfonic acid salt), followed by mounting in 3% methylcellulose was used prior to all ablations. For all ablations zebrafish were positioned laterally with their right side up.

### Laser ablation

Laser ablation was carried out using a MicroPoint® Ablation Laser System (Photonic Instruments, Arlington Heights, IL), which uses a nitrogen laser attached to a Nikon Eclipse 50i compound microscope. This technique was established by Berns et al., [24] and has been used to target cells without damaging underlying and surrounding tissues in various vertebrate systems (e.g. axolotl: [25,26]; zebrafish: [27,28]. Targeted cells are aligned using the microscope cross-hairs and ablated with a focussed laser beam which was fired at 2 Hz for 1 second, repeatedly. Prior to all laser ablation experiments, the laser beam was calibrated and the optimum beam size and firing rate was determined.

### Skeletogenic condensation ablation

Infraorbital five was selected for ablation since it develops second in the IO series at 10.0 mm SL (together with IO3), it has a small condensation (suitable for ablation) and it is also one of the smallest IO bones in the series [4]. Once transgenic fish reached approximately 8.0 mm SL they were regularly examined for the presence of the infraorbital five (IO5) condensations. Monitoring was conducted by anaesthetizing the fish, mounting in 3% methylcellulose as described above and viewing with a Nikon Eclipse 50i compound microscope (CY3 filter excitation: 530-560, emission: 573-648; Nikon Intensilight C-HGFI system). This starting time-point for monitoring was determined based on previous reports on the timing of IO bone formation [4,11]. As soon as the IO5 condensation was observed at around 8.0-11.0 mm SL, the fish underwent laser ablation. The condensation was considered ablated when fluorescence could no longer be observed in the IO5 condensation (N = 8) or anaesthetized control fish were mounted and observed under fluorescence (N = 7). Follow-up monitoring of individual specimens to observe the growth of the ablated IO5, the control-side IO5, and control specimen IO5 elements was conducted at multiple time-points: 24 hours, 5 days, two week, one month and two months post-ablation. Once surviving condensation ablation and control (unablated) specimens (N = 4 and N = 5, respectively) reached adulthood they were euthanized in 0.1% MS222 and fixed in 10% NBF (neutral buffered formalin) overnight at room temperature. Following fixation specimens were dehydrated through a graded ethanol series to 70% EtOH for storage.

### Infraorbital canal neuromast ablation

In order to determine whether IO canal neuromasts influence the ossification of canal walls, IO neuromasts were ablated at both larval and juvenile time-points. The larval ablation was done once the neuromast deposition by the neuromast primordium [12] was complete at 5 dpf (N = 20) [45]. The juvenile ablation was performed immediately before neuromasts have been suggested to induce canal wall ossification [13]. For both IO3 (N = 10) and IO5 (N = 20) ablations, this time-point was at 10.0 mm SL. Once zebrafish reached the desired size, they were vitally stained through immersion in 3 μM FM1-43X for 20 minutes in the dark at room temperature. Following staining, zebrafish were rinsed. Zebrafish were individually anaesthetized and mounted. Specimens were viewed using a Nikon Eclipse 50i compound microscope using FITC (Excitation: 460-500, Emission: 510-560) fluorescence emitted using a Nikon Intensilight C-HGFI system. The neuromasts to be ablated were then aligned with the ocular cross hairs and the laser was fired. This ablation was repeated until there were no fluorescing neuromast cells visible in the targeted region. Specimens were then removed from the methylcellulose solution, rinsed in fresh zebrafish system water and returned to a container of system water to recover. Following recovery, specimens were returned to their tanks in the fish facility. Control specimens (no ablation) (N = 20) were similarly stained, mounted and released.

Follow-up monitoring of individual specimens was conducted at 24 hours and at 3-5 days post-ablation by re-staining the specimens in FM1-43 as described above. Six months post-ablation (i.e. at adulthood) specimens were fixed in 10% NBF overnight at room temperature, dehydrated through a graded ethanol series to 70% and stored.

### Histological analyses of neuromast ablation

To evaluate whether the laser ablation technique damaged surrounding tissues, histological analyses were conducted comparing the neuromast after ablation to the unablated corresponding neuromast on the anterolateral side of the head. Zebrafish that underwent neuromast ablation were euthanized and fixed in 4% PFA in PBS overnight at 4°C at two time points; immediately following ablation (N = 5) and 3 days after ablation (N = 5)*.* Following fixation the heads were removed and embedded in paraffin wax (Fisher Scientific, Paraplast Xtra) according to standard procedures. Sections were cut at 5 μm, placed on APTES (aminopropyltriethyl saline) coated slides [46], and stored at 4°C. Masson’s Trichrome staining was conducted on sections as described in Franz-Odendaal et al., [47]. Additionally, apoptosis was detected using the TUNEL (terminal deoxynucleotidyl transferase-mediated deoxyuridinetriphosphate nick end-labeling) Detection kit (Roche, #11684817910) following the staining protocol by [48] with modifications by J. Jabalee (personal communication). All sections were viewed with a Nikon Eclipse 50i compound microscope.

### Analyses of adult infraorbital bones and canal mineralisation

To investigate the effects of both condensation and neuromast ablation in adult specimens, bone staining was conducted using the standard protocol for Alizarin Red [10,49]. Infraorbital bones were analyzed both *in situ* and following dissection using a Nikon SMZ1500 stereomicroscope and Nikon 50i eclipse compound microscope. Infraorbitals were photographed using a Nikon DXM 1200C camera and measurements were taken using Nikon NIS elements software. Once dissected infraorbital bones were photographed and prepared for shape analysis using the suite of SHAPE software [29]; http://lbm.ab.a.u-tokyo.ac.jp/~iwata/shape/). Shape analysis enables a quantitative evaluation of biological contour shape based on Elliptic Fourier Descriptors (EFD) [29]. Photographs of dissected infraorbitals were inserted into Microsoft PowerPoint 2010 and outlined using the trace function. Outlines were saved in full colour to a BitMap file. The digital outlines were then uploaded to the program ChainCoder where the contours of the outlines were extracted and stored as chain code [50]. The chain codes were then normalized into elliptical Fourier descriptors (EFDs) in the program Chc2Nef. Next, the program PrinComp performed principal component analysis on the EFDs. Principal component results were plotted using MYSTAT 12.02.00 [51] along with 95% confidence interval ellipses for all treatment groups (ablation, control and control-side). Finally, shape change accounted for by each resulting principal component was visualized using the program PrinPrint, this procedure was based on that of [52].

To evaluate the amount of infraorbital canal wall mineralization for neuromast ablation specimens, the length of raised canal walls on the targeted IO bone was measured using Nikon NIS software on high magnification photographs. Four measurements were taken during this procedure. The maximum length of the canal wall (i.e. where the canal wall will form) and the length of the section of the canal wall that was raised (approx. perpendicular to the IO bone) were measured for both walls of the canal on individual IO bones (namely IO3-IO5). From these measurements the percentage of canal wall mineralized was calculated using the following formula: (length of anterior raised canal wall plus length of posterior raised canal wall) divided by (length of anterior canal edge plus length of posterior canal edge) multiplied by 100. This ratio was calculated for each treatment (larval IO3 and IO4 neuromast ablation and for juvenile IO3 and IO5 neuromast ablation) and the control group (no ablation). These ratios of canal wall mineralization were compared using an independent samples t-test in SPSS Version 19 [53]. To visualize canal wall mineralization for all treatment groups, a grouped bar chart was constructed using R 3.1.0 [54] and R Studio [55].

## Key findings

- Skeletogenic condensations can recover from targeted ablation; however, their subsequent development is affected; adjacent elements are unaffected;
- IO bones possess two distinct semi-independent phases of development;
- Ablated neuromasts recover from ablation faster in juveniles compared to in larvae
- Neuromast ablation affects canal wall mineralization but not overall IO bone shape.
